# Rating experiments in forestry: How much agreement is there in tree marking?

**DOI:** 10.1371/journal.pone.0194747

**Published:** 2018-03-22

**Authors:** Arne Pommerening, Carlos Pallarés Ramos, Wojciech Kędziora, Jens Haufe, Dietrich Stoyan

**Affiliations:** 1 Forest Resource Management Department, Swedish University of Agricultural Sciences, Faculty of Forest Sciences, Umeå, Sweden; 2 Chair of Forest Management Planning, Faculty of Forestry, Warsaw University of Life Sciences, Warsaw, Poland; 3 Technical Development, Forest Research, Ae Village, United Kingdom; 4 Institut für Stochastik, Technische Universität Bergakademie Freiberg, Freiberg, Germany; Rice University, UNITED STATES

## Abstract

The process of selecting individual trees by humans for forest management purposes is the result of a plethora of factors and processes that are hard to disentangle. And yet in the past many textbooks and other publications have maintained that this selection leads to somewhat unanimous results. In this study, we analysed the data of 36 so-called marteloscope experiments from all over Britain, which are managed by the Ae Training Centre (Scotland, UK). Our objective was (1) to establish how much agreement there actually was when asking test persons (raters) to apply two different thinning methods, low and crown thinning. In addition we (2) were interested in understanding some of the processes leading to certain levels of agreement and in relationships between the agreement measures and characteristics of forest structure. Our analysis was based on multivariate statistics, particularly using Fleiss’ kappa. This was the first time that an analysis of rater behaviour was performed at such a large scale and it revealed that the general agreement in tree selection in Britain was only slight to fair, i.e. much lower than in medical experiments. The variability of selecting individual trees was considerable. We also found that agreement in tree selection was much stronger in low-thinning as opposed to crown-thinning experiments. As the latter is an important method of Continuous Cover Forestry and British forestry is increasingly adopting this forest management type, our results suggested that there is a need to provide more training. Interestingly the different levels of agreement as identified by Fleiss’ kappa could not be explained by measures of forest structure, however, the mean conformity number, a surrogate of Fleiss’ kappa, showed correlations and indicated that conformity increased with increasing complexity of tree stem diameter structure.

## Introduction

Modern forestry is essentially the result of two centuries of experimentation at practical and research level. Current forest management textbooks owe much to a historical evolution of forest practices, which is partly based on regional, cultural tradition and experience, partly on research evidence [1]. The latter stems from long-term monitoring and experiments and respective conclusions have been converted to silvicultural prescriptions and management guidelines [2, 3]. These often give vague, sometimes more detailed recommendations and there is a lot of freedom for machine operators and field staff to implement them in a way that personally seems most appropriate to them. This personal interpretation of prescriptions and research results naturally gives rise to much variation that has not been widely acknowledged and considered so far [4].

Thinnings are typical management operations where some trees are selected for removal to favour others that remain in the forest stand under consideration [5]. In this process, tree stems have to be physically marked (using ribbons or spray paint) for eventual removal and/or for long-term retention and this marking is typically based on the aforementioned prescriptions and guidelines. In some countries, the operators of harvester machines select trees for removal on an *ad hoc* basis as they are driving through the forest.

Until recently, forest managers and researchers have often assumed that this kind of tree marking leads to almost unanimous results with hardly any variation given that the staff in question had the same education and thinning instructions. Research starting in the 1990s has cast considerable doubt on this assumption [6, 7, 8]. Apparently there is much uncertainty in tree marking, which results in a considerable variation in the selection of trees.

When selecting trees, regardless of the management objective, a major decision is taken that will affect the dynamics of a stand for many years if not decades [9] to come. This is where the importance of selecting trees lies, i.e. the process is directly linked to management objectives and to the practical application of knowledge. A single management operation can severely affect the dynamics of a forest stand, since it is a disturbance in the natural development of a forest comparable to pathogen infestations and sporadic fire. It is possible to predict the growth and dynamics of a stand rather accurately [10, 11, 12] and then to simulate the consequences of alternative tree removals, for example, in terms of growth and yield [13, 14]. Research in tree selection agreement can effectively complement forest modelling by including person-specific tree selection behaviour and by quantifying the uncertainty of forest management introduced by tree selection.

The question of agreement between professionals judging an object is very common in medicine and part of assessing reliability and reproducibility of decision making as well as quality assurance in this field [15]. Studying agreement between individuals selecting trees has so far largely been neglected. Until now research in this field has focused on modelling and simulation of different thinning interventions and intensities [16, 17, 18, 19, 20, 21, 22] following the rationale of forest growth and yield experiments. The modelling work of these studies was mainly concerned with the objective of predicting forest dynamics including thinnings and harvesting. However, the modelling of these processes has always focussed on textbook or best-practice scenarios and hardly ever attempted to quantify the differences in forestry staff charged with the same task of marking trees.

With ongoing climate change, increasing importance of forest conservation and an emphasis on balancing ecosystem goods and services, the question of human tree selection behaviour has gained renewed attention.

Spinelli et al. [23] studied the silvicultural results (in terms of basal area and trees per hectare) performed by a number of test persons or raters with different professional backgrounds in mixed continuous-cover-forestry woodlands in Northern Italy. They found no significant difference in the marking behaviour of raters from different professional groups, however, they also identified a substantial lack of agreement in terms of the selection of individual trees.

Vítková et al. [24] could demonstrate that education and subsequent training can profile people’s choices in terms of tree selection behavior. The authors reported tree marking experiments involving raters with different experience and education. They required the raters to perform the marking twice in the same experimental forest, once before and once after training in crown thinning methods. Experts were unwilling to adopt the new thinning method and the training led to confusion and decreasing agreement in this group. In contrast, novices responded well to the training and the agreement in this group was significantly higher than among the experts.

Human tree selection research implies the collection of data from specific sites where a comparison between the behaviour of individuals is possible. To facilitate this, the marteloscope experiment was developed for monitoring human decisions in tree selection. In terms of layout and mensuration protocols, the marteloscope is similar to a standard forest research plot, where the data of all trees or of a subset of all trees within a bounded area are collected and recorded: stem diameter at breast height (1.3 meters above ground level), tree species, tree locations (Cartesian coordinates) and optionally some additional qualitative features like log quality and habitat suitability. In addition to these measurements, trees are labelled with numbers, for two purposes: (1) for the identification of trees in the field experiment and (2) for linking individual trees with measurements. Marteloscopes are often used for practical training in forestry, where trainees are required to mark trees according to some instructions and objectives [25, 26]. The trainees’ choices are then compared with those of experts or models, which are questionable references because of subjective or idealised conditions. Marteloscopes are becoming more and more popular, especially as tools for knowledge transfer and training activities [27].

As a potentially refined alternative to the marteloscope method, it is possible to subset the trees of a marteloscope with the objective to pre-select trees of particular importance. Such a subset typically does not include trees for which the decision-making process is trivial. According to our experience an agreement analysis of subsets produced in hindsight after the experiment, e.g. by removing all trees which were equally considered by all test persons or trainees, does not make sense. Naturally it is also possible to study human tree selection behaviour on small forestry inventory plots.

Marteloscope-based tree-marking training is applied in a number of European countries and also in the United States and Canada (see Table 1). The technique is possibly also used in some Asian and South American countries; however, it is difficult to identify relevant information from those regions.

**Table 1:** Projects and organisations involving the use of marteloscopes for in-situ tree-selection training.

Project/Organisation	Location/Country	Objective	Website/URL
AFI (Association Futaie Irrégulière)	France	Monitor Continuous Cover Forestry (CCF)	http://prosilva.fr/html/index.html
CPFC (Centre de la Propietat Forestal de Catalunya)	Barcelona, Spain	Forestry training	http://cpf.gencat.cat/en/index.html
CRPF Auvergne (Regional Center of Private Forest Property of Auvergne)	France	Forestry training for CCF	http://www.crpfauvergne.fr
Forestry Commission UK	United Kingdom	Forestry training for CCF	http://www.forestry.gov.uk
Joseph W.Jones Research Center	Georgia, United States	Forestry training for multi-aged forest stands	http://www.jonesctr.org
SelectFor	Wales, United Kingdom	Forestry training for CCF	http://www.selectfor.com
Silvicultural Competence Centre	Lyss, Switzerland	Forestry training for CCF	http://www.waldbau-sylviculture.ch/94_martelo_d.php
University of Lleida	Lleida, Spain	Forestry training	http://www.forestal.udl.cat/es
University of Moncton	Canada	Forestry training	https://www.umoncton.ca
University of Valladolid	Valladolid, Spain	Research and forestry training	http://www.uva.es/export/sites/uva/
Warsaw University of Life Sciences	Warszawa, Poland	Forestry training	http://en.uw.edu.pl
Hammer Project	France, Finland, Italy, Belgium and Spain.	Build a digital platform for thinning simulations	http://www.hammer-project.eu
Integrate+ (EFI, EFICENT & BMEL)	Freiburg, Germany	Create a European network of demonstration sites and specific software to be used in portable devices.	http://www.integrateplus.org

AFI (Association Futaie Irrégulière) together with the AgroParisTech-ENGREF at Nancy (France) were the first organisations that put forward the idea of marteloscopes as a tool for monitoring human tree selection behaviour [28, 26]. AFI, CRPF (Regional Centre of Private Forest Property), CPFC (Centre de la Proprietat Forestal de Catalunya) and a number of universities (see Table 1) regularly organise marking exercises with students, forest stakeholders (private forest owners, forest managers, forest workers) and with members of the general public including an evaluation and a discussion of individual tree selection performance. The Forestry Commission in the United Kingdom maintain a forest training centre (Ae Training Centre, Scotland) that regularly offers silvicultural and other training courses. These include tree quality assessments of timber and recreational aspects and also the marking of trees for conservation. The marking exercises also include an individual evaluation of tree markings as well as comparisons between course participants. In addition, the British company SelectFor offers services related to the installation of marteloscopes and to forestry training in CCF based on marteloscope exercises. They have established marteloscope networks mostly in Wales and Ireland. The Joseph W. Jones Research Center in Georgia (USA) conducts workshops on managing longleaf pine (*Pinus palustris* Mill.) including tree marking exercises for obtaining an irregular forest structure. The variability between individuals in relation to the marking process is discussed during these exercises. In the *Hammer* and *Integrate+* projects specific software was developed that can be installed on portable devices such as tablets and allows electronic data entry in the field and an immediate analysis. Finally, the Swiss Silvicultural Competence Centre (Fachstelle Waldbau–Centre de compétence en sylviculture) in Lyss maintains a network of marteloscopes in all major forest types of Switzerland, which are regularly re-measured and actively used for educational courses in forest management offered to forestry students as well as to professionals. Also here each participant’s choices is analysed and documented by a dedicated analysis spreadsheet [29].

In this study, we analysed and discussed the agreement among individuals marking trees for thinnings. The objective of this study is to understand how much agreement is there between humans marking trees for different purposes, which processes and factors affect agreement and whether the level of agreement is related to structural properties of the forests where the marking takes place. For this purpose we analysed data from twelve sites including 36 experiments in Britain using different agreement indices. This is the first time that a systematic study involving such a large number of experiments covering a whole country was ever performed. In this meta-analysis, we always considered collectives of raters and were interested in the homogeneity of these collectives. We also considered relationships between agreement measures and characteristics of forest structure.

## Materials and methods

### Study sites

**Fig 1:**
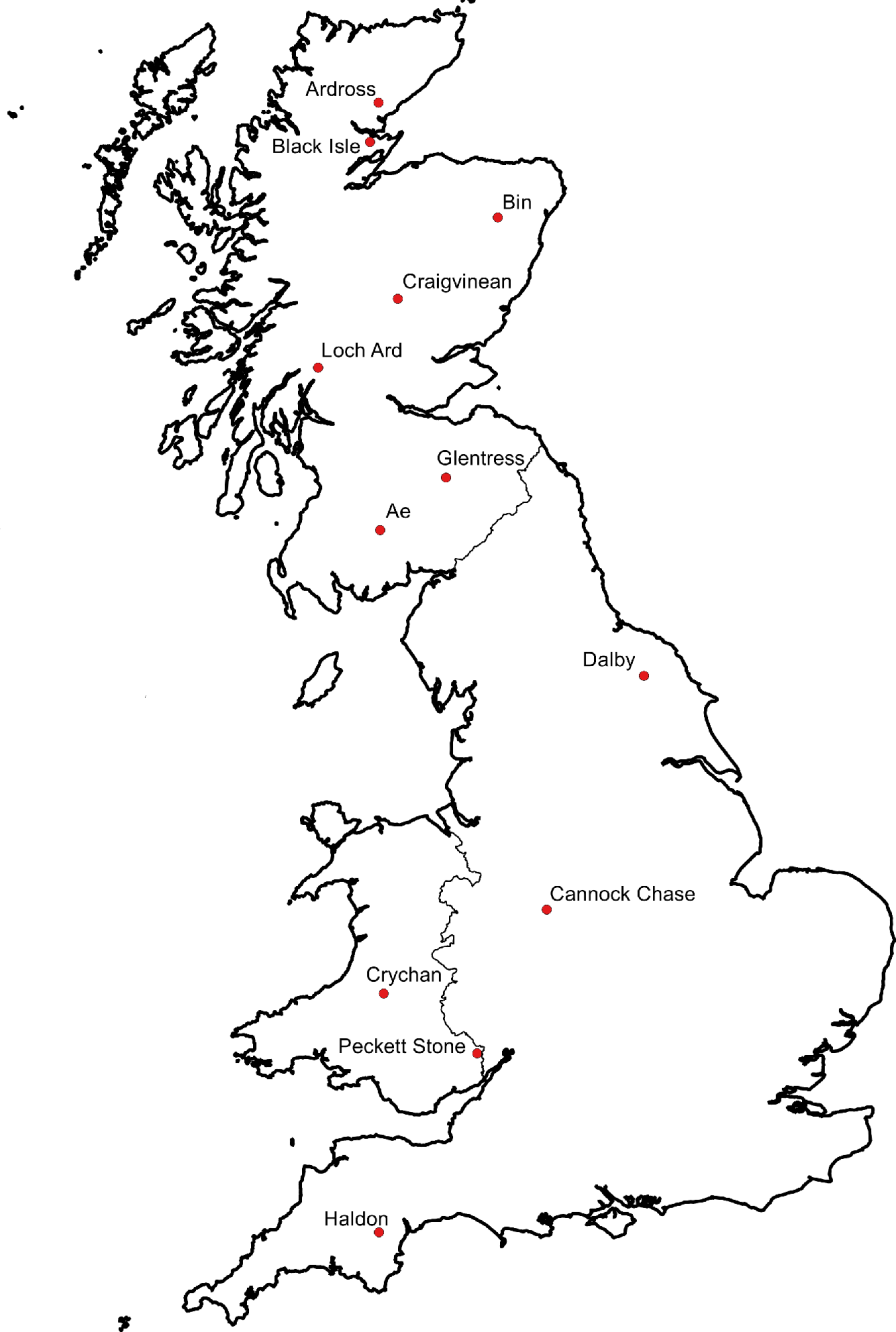
Location of the UK marteloscope sites considered in this study.

For this study, data from twelve marteloscope sites managed by the Ae Training Centre (Scotland, UK) were analysed. On all sites there was a considerable thinning urgency providing sufficient incentives for tree marking. The sites are widely distributed in Great Britain as shown in Fig 1.

Most of the sites include plantations of Sitka spruce (*Picea sitchensis* (Bong.) Carr.), hybrid larch (*Larix × marschlinsii* Coaz), Japanese larch (*Larix kaempferi* (Lamb.) Carr.) and Scots pine (*Pinus sylvestris* L.). In some of these stands, other species have later colonised the site, but the aforementioned species represent the main species in terms of density. Peckett Stone at the Welsh-English border is a beech (*Fagus sylvatica* L.) forest and therefore the only exception from the aforementioned species composition.

Each marteloscope had a size of 0.1 hectares and for each tree the following variables were measured: diameter at breast height (*d*) (measured in centimetres at 1.3m height), total tree height [m] and Cartesian coordinates in metres. We calculated basic summary characteristics and presented them in Table 2.

**Table 2:** Description of the sites included in this research. *N* (density, calculated as number of trees per hectare), *G* (basal area, calculated as the sum of the cross-sectional tree stem areas at breast height), *dg* (quadratic stem diameter at breast height), *h*_100_ (stand top height calculated as the mean height of the 100 largest trees per hectare), *v*_*d*_ (coefficient of variation of stem diameters at breast height) and *k*_*d*_ (skewness of the empirical stem diameter distribution).

Site	Species	*N* [trees/ha]	*G* [m^2^/ha]	*dg* [cm]	*h*_100_ [m]	*v*_*d*_	*k*_*d*_
Ae	Sitka spruce	1336	41.9	20.1	21.1	0.35	0.17
Ardross	Hybrid larch	2180	32.3	13.7	13.4	0.37	0.49
Bin	Sitka spruce	1540	59.3	22.1	22.1	0.30	0.12
Black Isle	Scots pine	2010	26	12.8	10.8	0.24	0.18
Cannock Chase	Hybrid larch	2040	36.7	14.9	14.6	0.29	0.07
Craigvinean	Sitka spruce	3000	56.7	15	14.8	0.24	0.07
Crychan	Hybrid larch	1930	41.2	16.5	16.1	0.28	-0.04
Dalby	Japanese larch	1900	46.2	17.6	18.6	0.28	0.31
Glentress	Sitka spruce	1760	58.1	20.5	23.2	0.29	0.06
Haldon	Sitka spruce	1780	43.9	17.7	18.6	0.35	0.39
Loch Ard	Sitka spruce	2450	43.3	15	17.9	0.35	0.36
Peckett Stone	Beech	830	34.7	23.1	24.5	0.29	0.33

All sites had very high tree densities both in terms of trees per hectare and basal are per hectare. Stem size diversity as described by the coefficient of variation is rather low, which is typical of plantations that have received little management compared to for example forest stands managed according to the principles of near-natural forest management [30].

In most cases experiments involving low and crown thinnings were conducted with the same raters in the same marteloscope sites and some of the experiments were repeated in subsequent years, which contributed data from a total of 36 experiments to this study.

### Raters

The study included nineteen groups of test persons rating the trees as part of training sessions. In the statistical literature, such test persons are referred to as raters (see for example [31]) and we use this term in the remainder of our paper. Each group was comprised of a number of raters varying from a minimum of 9 to a maximum of 20. About 95% of the raters were employed by the state forestry service (Forestry Commission, Natural Resources Wales) in different capacities ranging from machine operators to work supervisors and also included woodland officers and forest managers. The remaining 5% of the raters mainly worked as forestry contractors.

In general terms, we considered the case where *r* raters classify *n* trees. All raters rated all trees by filling in questionnaire-like marking sheets. The binary classification involved two categories, i.e. “0” (negative–not selected) and “1” (positive—selected). The system of *r* × *n* marks “0” and “1” constituted the data and they were analysed for similarity in rating behaviour. This is a problem that has, as previously mentioned, so far only rarely been considered in forest science, but is quite common in medicine, psychology and sociology [32, 33, 34].

### Experiments and data structure

The experiments conducted on each site included two different thinning types. The first experiment involved a low thinning, otherwise known as thinning from below, where trees are removed mainly from the lower canopy and from among the smaller diameter trees [5]. The main objective of this type of thinning is to promote the growth of larger trees by removing smaller ones. The second type of experiment involved a crown thinning, also referred to as thinning from above, where trees are removed that are part of the main canopy in order to favour the best trees of the main canopy by removing their direct competitors [5]. The raters were provided with specific thinning instructions, which slightly varied from site to site depending on local conditions.

We note here that it is the objective of our paper to study the agreement of the whole collective of raters only. If a statistician would find a high degree of agreement, then no additional analysis of the rater behaviour is justified. Otherwise, there is some specific pattern in the collective, e.g. there may be subgroups of different agreement. Then one could try to find an explanation for the different rating behaviour and look for cause-and-effect relationships.

### Active and passive rating behaviour

In the experiments considered in this study, rating is influenced by two processes, an *active* and a *passive* process: (1) the rater activity performed from the point of view of the raters. A simple indicator of this activity is the number of marks given by a single rater. There may be raters that mark many and others that mark only few trees.

The second process (2) is the *passive* attraction evoked by the trees. A simple indicator of this passive process is the number of raters selecting a given tree. There may be trees where the decision is clear, whilst for others it is much less obvious even to experts.

A first natural step to understand these two processes is to use bar charts. They represent the marginal distributions of the rating data matrix and give valuable information on the rating behaviour.

For process (1), depicting the rater activity we can create a chart showing the proportions *n*_*i*_ / *n* of trees selected, where *n*_*i*_ is the number of trees selected by rater *i*. Clearly, this results in *r* bars and we refer to this bar chart as the *rater bar chart*.

The passive marking frequency of the trees (process 2) can be analysed by a bar chart showing the proportions of *k* / *n* of trees selected, where *k* is the number of marks “1” assigned by different raters with *k* = 0, 1, …, *r*. With *r* raters there are potentially *r* + 1 bars, as trees can also be selected by no rater. To this bar chart we refer to as the *marking bar chart*.

The two processes interact in a complicated way. Therefore it is difficult to disentangle them and to characterise their joint effects by simple statistical characteristics.

### Statistical measures of interrater agreement

We studied the question of agreement among the raters, i.e. whether there is any agreement at all among the raters and if so to which degree have the raters arrived at similar conclusions. We note that for two raters (*r* = 2) agreement simply means a high number of equal decisions while for more than three raters agreement is difficult to describe.

There is a standard characteristic for measuring the degree of agreement in a collective of *r* raters (with *r* > 2) and this characteristic is referred to as Fleiss’ kappa, *k*, [32, 31], which is frequently used in applied statistics. However, in [35] it was shown that kappa has its weaknesses. As we will see below it is of a strongly passive nature. Therefore we considered alternative characteristics of rater agreement such as Cochran’s *Q* test, the test statistic of the *χ*^2^ goodness-of-fit test of the hypothesis of a uniform distribution of active rating numbers, the mean correlation coefficient and the coefficient of variation, which can be calculated from the correlation matrix that contains the correlation coefficients of the rating results of all pairs of raters. However, our test calculations and comparisons have convinced us that these characteristics are not useful for agreement evaluation. But we nevertheless applied some other statistics that will be explained below.

#### Fleiss’ kappa

The concept of kappa is based on pairwise comparisons and has its roots in the one-way analysis of variance. Fleiss’ kappa can be expressed in different equivalent forms, which highlight various aspects of the nature of this statistical characteristic. The first form is given in Eq (1).

$$\kappa =\frac{p_{0}-p_{e}}{1-p_{e}},$$ (1)

where *p*_0_ is the observed proportion of ratings in agreement and *p*_*e*_ is the expected proportion of ratings in agreement. The formula for *p*_0_ is given by

$$p_{0}=\frac{2}{r\left(r-1\right)}\sum_{i=1,j>i}^{r}e_{ij}.$$ (2)

Here *e*_*ij*_ is *n*_*ij*_ / *n*, where *n*_*ij*_ is the number of trees which were assigned the same mark (“0” or “1”) by both raters *i* and *j*. Eq (2) shows one aspect of the nature of kappa: *p*_0_ is a mean closely related to agreement in pairwise comparisons. For the second term, *p*_*e*_, [32] set

$$p_{e}=p^{2}+{\left(1-p\right)}^{2},$$ (3)

where *p* = *N*_1_ / *nr* and *N*_1_ is the total number of marks “1” given in the experiment. The second form Fleiss’ kappa can take is

$$\kappa =1-\frac{1/n\sum_{j=1}^{n}s_{j}(r-s_{j})}{r\left(r-1\right)p(1-p)},$$ (4)

where *s*_*j*_ is the number of marks “1” of tree *j*. The term *s*_*j*_(*r* – *s*_*j*_) is a good choice for characterising agreement, as it takes extreme values for the cases *s*_*j*_ = *r* / 2 and *s*_*j*_ = 0 or *s*_*j*_ = *r*. See [35] for a proof of the equivalence with Eq (1). We see that *k* only depends on the *s*_*j*_’s, i.e. on the passive rating frequencies of the trees. This demonstrates the strongly passive nature of kappa. Therefore it makes sense to consider an alternative to kappa. Indeed, several authors [36, 37, 38] had the idea to replace *p*_*e*_ given by Eq (3) by

$$p_{e}=\frac{2}{r\left(r-1\right)}\sum_{i=1,j>i}^{r}\left(P_{i}P_{j}+\left(1-P_{i}\right)(1-P_{j})\right),$$ (5)

where *P*_*i*_ = *n*_*i*_ / *n*. Thus we obtain another kappa that we denote by *κ*_*CHS*_ [35], referring to the three original authors Conger, Hubert and Schouten. It can be assumed that *κ*_*CHS*_ includes more information on the active rating behaviour than *κ*_*F*_. However, a comparison of the two equations for *p*_*e*_ (Eqs 3 and 5) shows that the same kappa values are obtained, if the raters rate with equal activity, i.e. if the *P*_*i*_ values are equal to *p*.

As far as the interpretation of kappa is concerned, Landis and Koch [39] were the first to suggest guidelines for interpreting *k*, which were revised by Stoyan et al. [35], see Table 3, and relate to both kappa measures.

**Table 3:** Interpretation of *k* values proposed by Stoyan et al. [35].

*κ*	Interpretation
< 0.10	Poor agreement
0.10–0.33	Slight agreement
0.33–0.50	Fair agreement
0.50–0.67	Moderate agreement
0.67–0.90	Substantial agreement
≥ 0.90	Almost perfect agreement

#### Other parameters

A number of parameters can be derived from the two bar charts and the empirical distributions they describe. These parameters help to characterise rater agreement.

Based on previous work [40] we applied the mean conformity number, $\bar{c}$. This characteristic quantifies the mean tendency of the raters to conform with the general rating tendency of all raters. The conformity of the rating result of rater *i* with those of the other raters is characterised by the conformity number *c*_*i*_. This is the mean of the numbers of raters who also selected the trees selected by rater *i*,

$$c_{i}=\frac{1}{n_{i}}\sum_{j=1}^{n}1_{ij}\cdot s_{j}\;\mathrm{for}\;i=1,2,\ldots ,r,$$ (6)

where *n*_*i*_ is the number of trees marked by rater *i* with “1” and *s*_*j*_ is the number of marks “1” of tree *j*. **1**_*ij*_ has a value of 1, if rater *i* marks tree *j* with “1”, otherwise the value is 0. The characteristic *c*_*i*_ takes large values, if rater *i* selects the trees selected by the majority of raters. $\bar{c}$ is the arithmetic mean of all *c*_*i*_ for a given experiment.

We also considered the coefficient of variation, *r*_*v*_, of the proportions of the rater bar chart. One of the parameters derived from the marking bar chart is the proportion of trees marked “0” by all raters, *P*_0_. This proportion constitutes a kind of “negative agreement” on “unselectable” trees. It typically includes trees that even to the eyes of a layman suggest the risk of worsening stand conditions in terms of silviculture, ecosystem goods and services as well as biodiversity, if they are removed from the forest. Matonis et al. [41] found evidence that humans tend to reach consensus much easier on negative than on positive choices. Therefore we included *P*_0_ in the list of parameters derived from the bar charts.

A complementary characteristic from the marking bar charts is the proportion of trees marked in the 20% highest classes of the marking bar chart, *P*_m_. We expected to gain insights from this characteristic that kappa does not yield.

For all these agreement parameters the statistical relationships with the characteristics of the following two sections can be analysed, which contributed to a better understanding of the rating process studied here.

### Tree size characteristics

One of our main questions in this study related to whether the tree characteristics of the forest sites might have had an influence on the different levels of agreement. We started with a longer list of possible parameters and after an initial screening reduced them to the best performing characteristics.

We considered the coefficient of variation, *v*_*d*_, of tree stem diameters and the skewness of the empirical diameter distribution, *k*_*d*_, as representatives of tree size variables. Both are often used as convenient characteristics of tree size diversity and forest stand structure, see Hui and Pommerening [42]. We expected that agreement would decrease with increasing structural complexity as quantified by these characteristics, because complexity may make the decision process more difficult.

Finally we included the ratio of the proportion of number of trees (*N*) marked with “1” and the proportion of basal area (*G*, derived from stem diameter using the area equation of the circle) of these trees [43] in the analysis, see Vítková et al. [24].

$$B=\frac{\mathrm{Proportion}\;\mathrm{of}\;\mathrm{the}\;\mathrm{number}\;\mathrm{of}\;\mathrm{trees}\;\mathrm{selected}}{\mathrm{Proportion}\;\mathrm{of}\;\mathrm{the}\;\mathrm{basal}\;\mathrm{area}\;\mathrm{of}\;\mathrm{selected}\;\mathrm{trees}}=\frac{P_{N}}{P_{G}}$$ (7)

This measure quantifies the tree selection strategy by comparing numbers of trees selected with their cumulative size. If *B* < 1, a smaller proportion of trees has been selected compared to their proportion of cumulative basal area. In a thinning context, this typically indicates a crown thinning and the trees selected show a tendency of being in the upper part of the empirical diameter distribution. A larger proportion of trees is selected compared to their proportion of basal area, if *B* > 1. In a thinning context, this is consistent with a thinning from below and trees were preferably selected in the lower part of the empirical diameter distribution. For each experiment, $\bar{B}$ was calculated as the arithmetic mean of all raters.

All calculations were performed using R [44].

## Results

### Bar chart analysis

**Fig 2:**
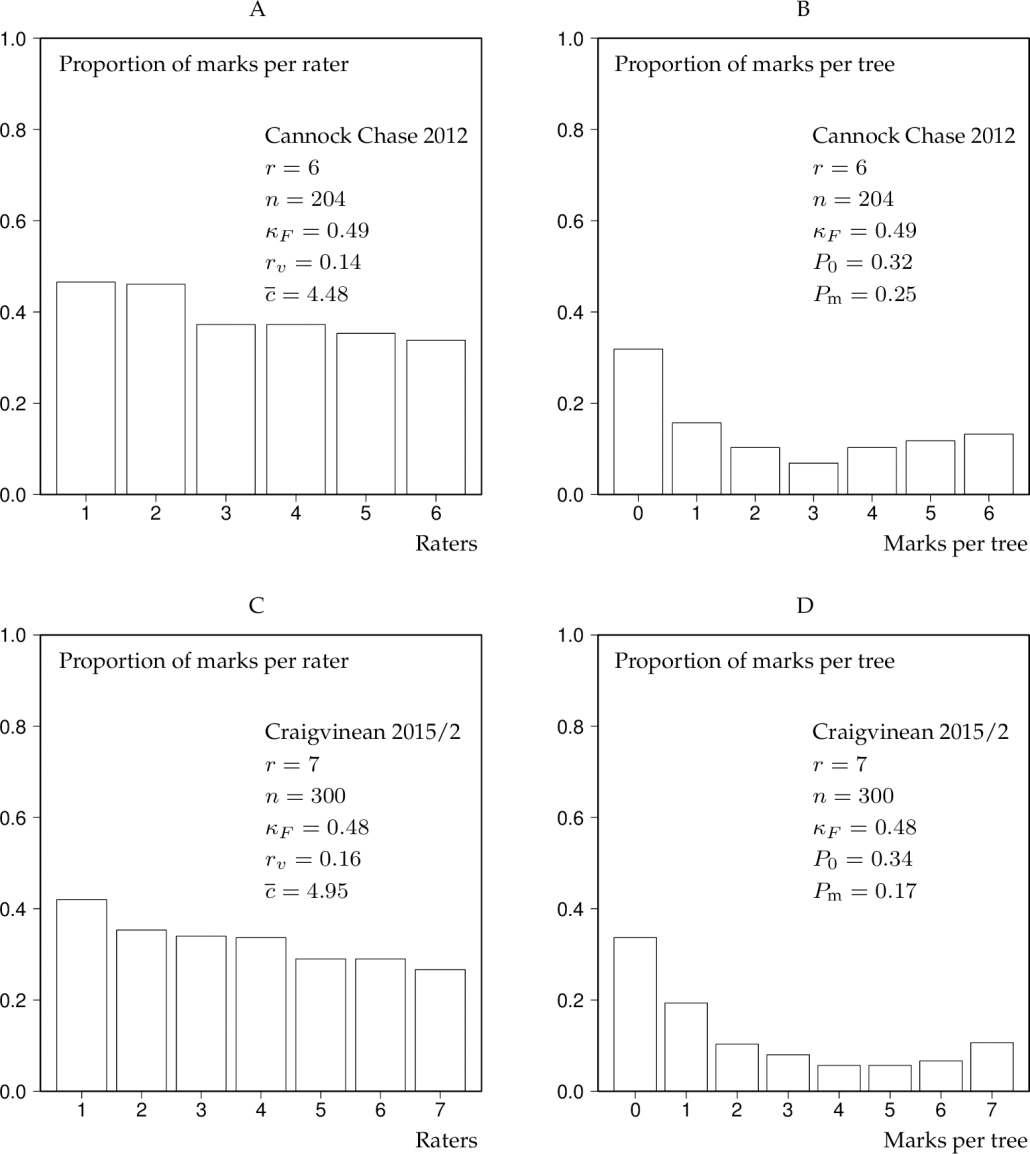
Rating and marking bar charts for Cannock Chase 2012 and Craigvinean 2015/2 as a result of low thinning experiments. The bars of the rater bar charts were ranked according to rating activity. *r*–number of raters, *n*–number of trees, *κ*_*F*_ –Fleiss’ kappa, *r*_*v*_−coefficient of variation of the proportions of the rater bar chart, $\bar{c}$–mean conformity number, *P*_0_–proportion of trees marked “0” by all raters, *P*_m_−proportion of trees marked in the 20% highest classes of the marking bar chart, see Section “Statistical measures of interrater agreement”.

**Fig 3:**
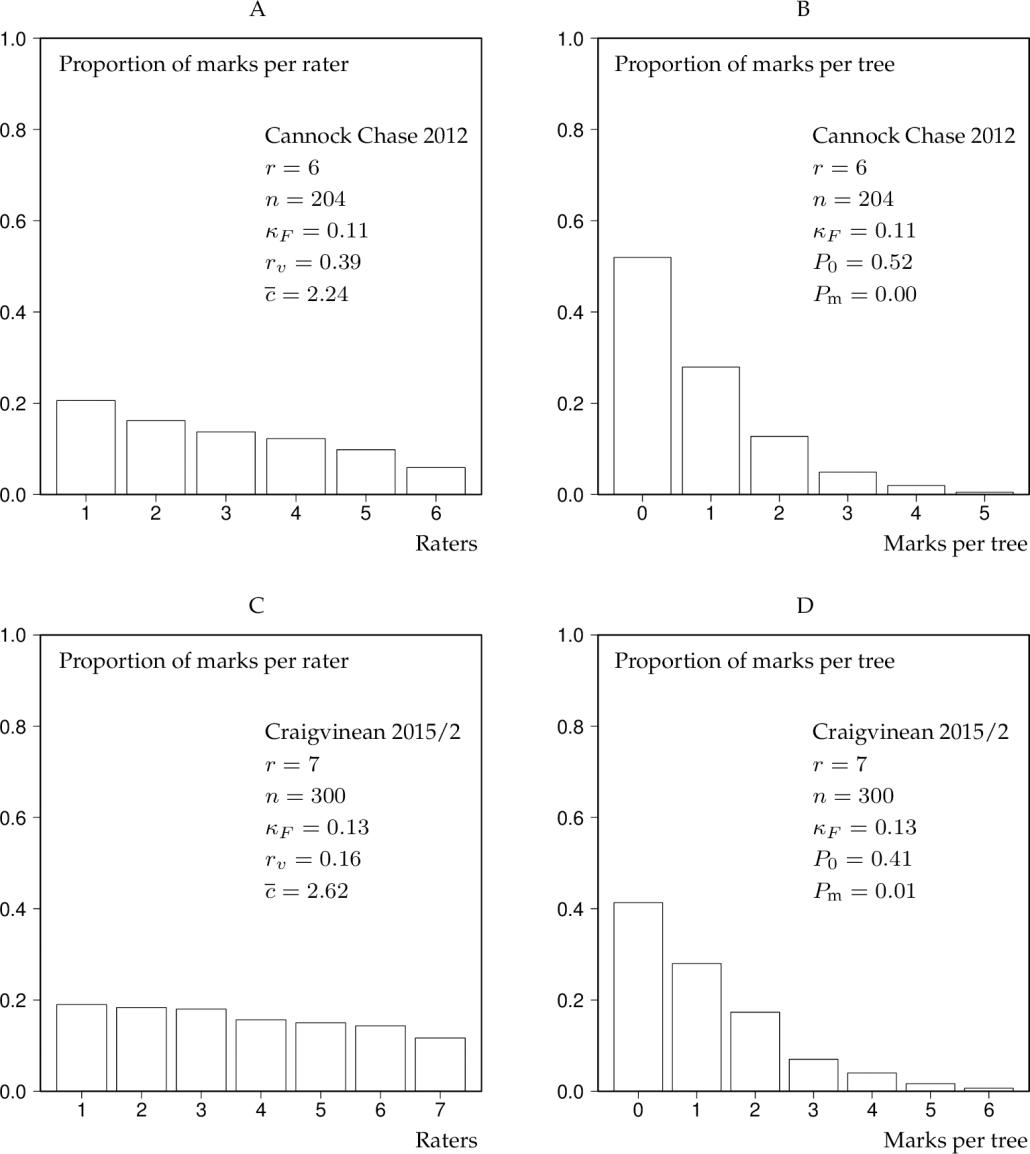
Rating and marking bar charts for Cannock Chase 2012 and Craigvinean 2015/2 as a result of crown thinning experiments. The bars of the rater bar charts were ranked according to rating activity. *r*–number of raters, *n*–number of trees, *κ*_*F*_–Fleiss’ kappa, *r*_*v*_−coefficient of variation of the proportions of the rater bar chart, $\bar{c}$–mean conformity number, *P*_0_ –proportion of trees marked “0” by all raters, *P*_m_−proportion of trees marked in the 20% highest classes of the marking bar chart, see Section “Statistical measures of interrater agreement”.

As examples demonstrating the value of the bar charts we here present the results of the analysis Cannock Chase 2012 and Craigvinean 2015/2 for low thinning (Fig 2) and crown thinning (Fig 3).

In the case of low thinning, both marteloscope experiments lead approximately to the same “fair agreement” (Table 3). Also the bar-chart parameters *r*_*v*_, $\bar{c}$, *P*_0_ and *P*_m_ have similar values in both cases (Fig 2). When the same raters selected trees for a crown thinning, the situation was different (Fig 3): The statement suggested by *κ*_*F*_ reduced to “slight agreement”, the *r*_*v*_ value for Cannock Chase 2012 (Fig 3A) much increased and the values of $\bar{c}$ have markedly decreased. The proportions of marks per rater were closer to a uniform distribution in the case of Craigvinean 2015/2 (Fig 3C) compared to Cannock Chase (Fig 3A), hence the lower *r*_*v*_ value for Craigvinean 2015/2. *P*_m_ for both experiments was close to 0 (Fig 3B and 3D). Interesting is also the comparison between the marking bar charts in Fig 3: *P*_0_ in the marking bar charts of Figs 2B and 2D and 3B and 3D is represented by the first bar on the left labelled “0” whereas *P*_m_ is represented by the last two bars on the right in the marking bar charts of Figs 2B and 2D and 3B and 3D. In Fig 3B and 3D, the last bars for *k* = *r* are actually missing, i.e. their proportions are 0, hence *P*_m_ is near 0. Largely due to the typical *P*_0_ and *P*_m_ values, marking bar charts in low thinning experiments tended to be U-shaped (Fig 2B and 2D), whilst those related to crown thinnings (Fig 3B and 3D) mostly had an exponential shape. Comparing the bars in the rater bar charts of the low thinning experiments (Fig 2A and 2C) with those related to the crown thinning experiments (Fig 3A and 3C) interestingly reveals the much reduced rater activity in crown thinnings compared to low thinnings. This is consistent with the concept that fewer but larger trees are selected in crown than in low thinnings.

### Agreement analysis

**Fig 4:**
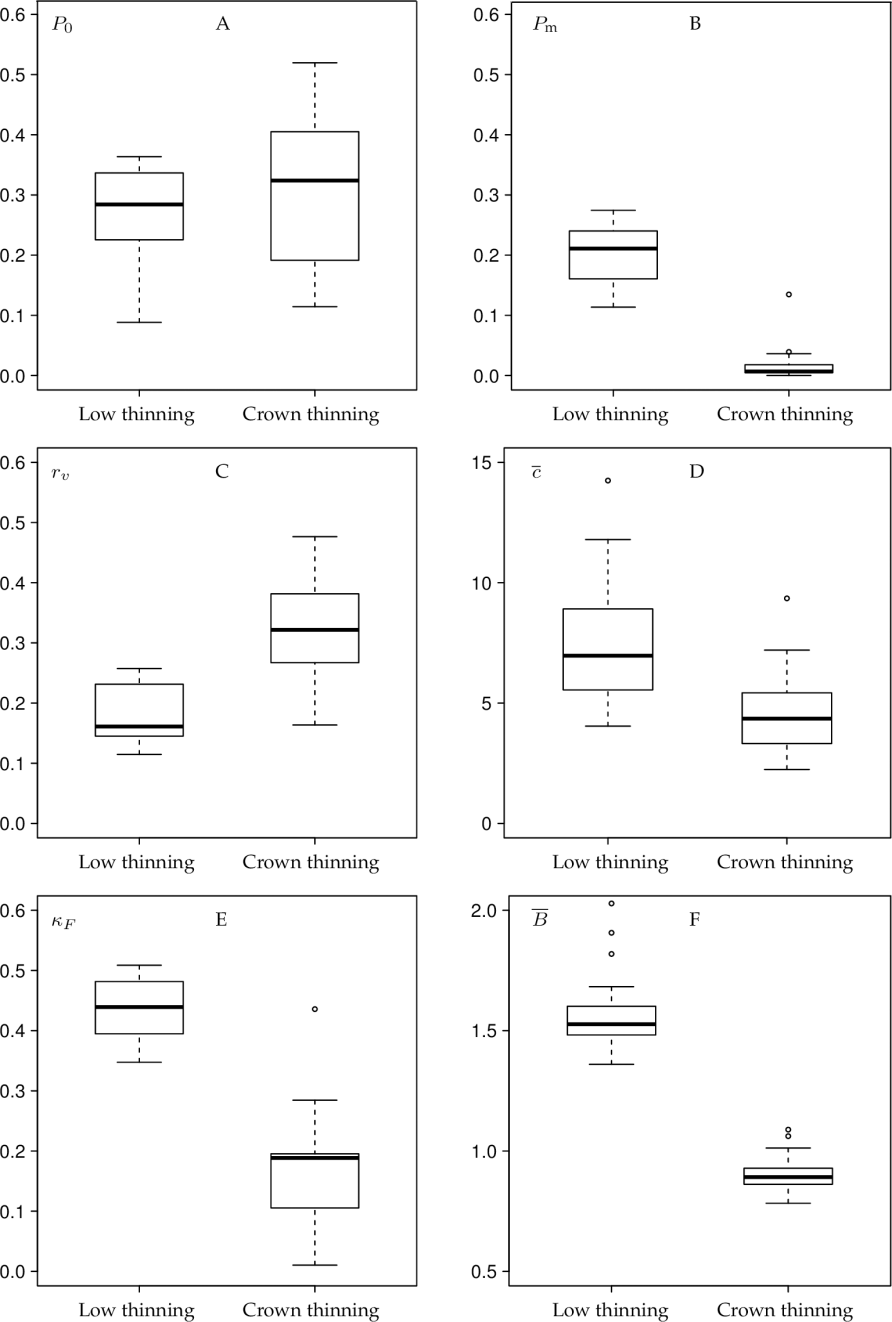
Box plots depicting the empirical distribution of the bar-chart parameters, Fleiss’ kappa and the mean ratio $\bar{B}$. *P*_0_ –proportion of trees marked “0” by all raters, *P*_m_−proportion of trees marked in the 20% highest classes of the marking bar chart, *r*_*v*_−coefficient of variation of the proportions of the rater bar chart, $\bar{c}$–mean conformity number, *κ*_*F*_ –Fleiss’ kappa, $\bar{B}$ - mean ratio of the proportion of number of trees marked with “1” and the proportion of basal area of these trees, see Section “Statistical measures of interrater agreement”.

Depicting the distribution of the bar-chart parameters, Fleiss’ kappa and the characteristic $\bar{B}$ as box plots separately for low-thinning and crown-thinning experiments revealed significant differences in the behaviour of the same raters when selecting trees for different thinning types (Fig 4). The only exception was parameter *P*_0_ (Fig 4A, *p* > 0.05), although one can argue that the parameter has a larger variance in crown thinnings. The ratio of trees selected by most raters, *P*_m_ (Fig 4B), was close to zero in crown thinnings and significantly higher in low thinnings (*p* < 0.001).

The rater activity is significantly more homogeneous in low than in crown thinnings as indicated by *r*_*v*_ (Fig 4C, *p* < 0.001). In a similar way the mean conformity number $\bar{c}$ (Fig 4D) and the Fleiss kappa characteristic *κ*_*F*_ (Fig 4E) show significantly more agreement in low thinnings than in crown thinnings (*p* < 0.001 for both). Finally, as a control, the ratio of the proportion of number and basal area of selected trees, $\bar{B}$ (Fig 4F), also clearly and significantly (*p* < 0.001) highlights the difference between the rating for the two thinning types. However, we can see here that the median of the crown-thinning experiments was quite high (close to 1), indicating that the raters were not too comfortable with the new thinning method and rather tended to fall back to traditional practices. Apart from *P*_0_ all medians were significantly different according to the paired *t* test.

### Relationships

**Fig 5:**
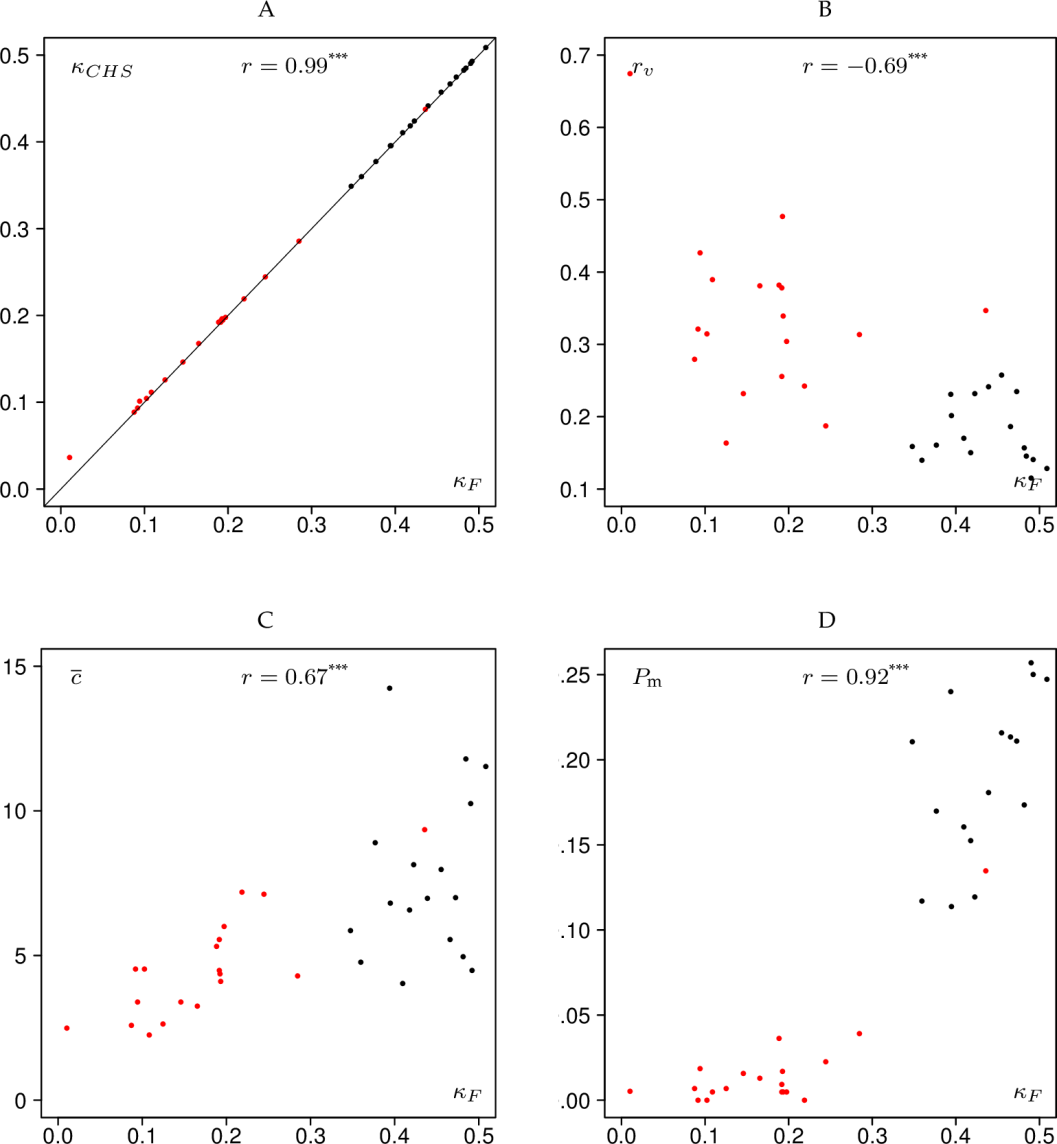
Scatter plots involving Fleiss’ kappa and three bar chart parameters. *κ*_*CHS*_–Conger-Hubert-Schouten kappa, *κ*_*F*_–Fleiss’ kappa, *r*_*v*_−coefficient of variation of the proportions of the rater bar chart, $\bar{c}$–mean conformity number, *P*_m_−proportion of trees marked in the 20% highest classes of the marking bar chart, see Section “Statistical measures of interrater agreement”.

**Fig 6:**
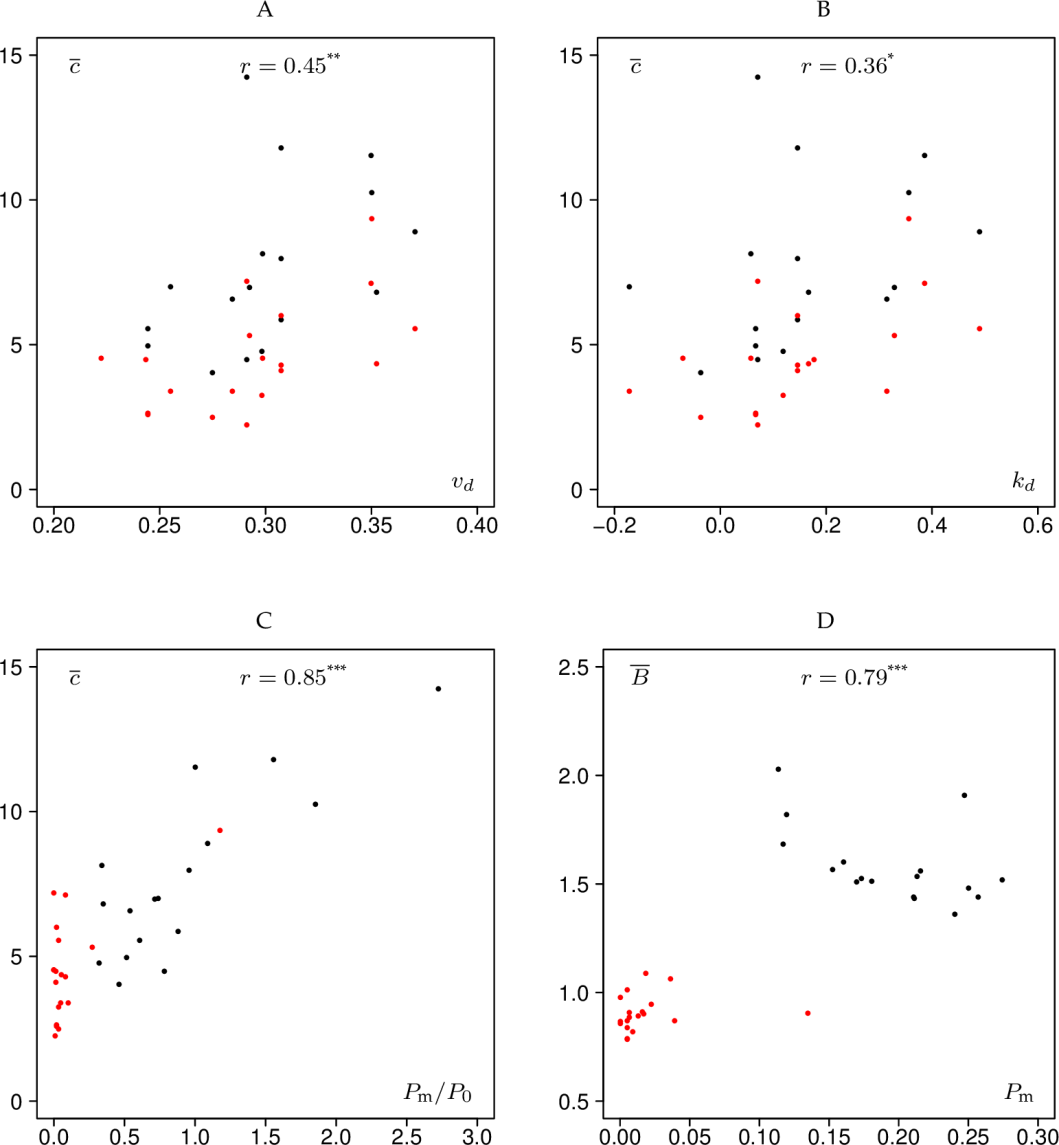
Scatter plots involving forest and bar chart characteristics. *r* denotes the Pearson correlation coefficient. “***” indicates *p* values < 0.001 and “*” indicates *p* values between 0.01 and 0.05. Black–low thinning, red–crown thinning. $\bar{c}$–mean conformity number, *v*_*d*_−coefficient of variation of stem diameters, *k*_*d*_−skewness of the empirical diameter distribution, *P*_0_ –proportion of trees marked “0” by all raters, *P*_m_−proportion of trees marked in the 20% highest classes of the marking bar chart, $\bar{B}$ - mean ratio of the proportion of number of trees marked with “1” and the proportion of basal area of these trees, see Section “Statistical measures of interrater agreement”.

In this section, we have explored the relationship between different variables using Pearson’s correlation coefficient. However, in some cases the relationships are nonlinear and inhomogeneity effects resulting from the application of the two thinning methods render the use of correlation coefficients questionable, although we have provided all values of Pearson’s correlation coefficients along with the levels of significance in Figs 5 and 6. Therefore in this section we rather stress the visual impression gained from the scatter plots.

### Relationships involving Fleiss’ kappa

Obviously there was a very strong correlation between *κ*_*F*_ and *κ*_*CHS*_, as expected, since the two characteristics are closely related, see Fig 5A Only one red data point (experiment Crychan 2010) did not lie on the 45° line. For the low thinning method, *κ*_*F*_ values generally were between 0.35 and 0.51 (fair to moderate agreement, see Table 3), while for the crown thinning method *κ*_*F*_ values were in a range between 0.01 and 0.28 (poor to slight agreement, with the notable exception of experiment Loch Ard 2015, here *κ*_*F*_ = 0.44).

*κ*_*F*_ was negatively related to *r*_*v*_, the coefficient of variation of the proportions of the rater bar chart (Fig 5B). The variation of rater activity increased with decreasing agreement. Fleiss’ kappa was positively related to the mean conformity number, $\bar{c}$. Conformity increased with increasing agreement (Fig 5C).

Finally *κ*_*F*_ and the proportion of trees marked by most of the raters, *P*_m_, were positively related in a nonlinear fashion (Fig 5D). In all charts, the colours also reveal the strict separation between low-thinning and crown-thinning experiments, which is only violated by one data point (the aforementioned experiment Loch Ard 2015). The use of the two thinning methods obviously introduces strong inhomogeneity effects. *κ*_*F*_ was tested for a significant difference from zero. All kappa values were indeed different from zero with the exception of Crychan 2010.

### Relationships involving forest characteristics

We hoped to identify a relationship between *κ*_*F*_ and forest characteristics, but interestingly none of them were significant. Instead we found a weak correlation between the mean conformity number $\bar{c}$ and the coefficient of variation of stem diameters, *v*_*d*_, (Fig 6A) and the skewness of the empirical diameter distribution, *k*_*d*_, (Fig 6B) respectively. These correlations suggest that conformity increases with increasing complexity of tree stem diameter structure. In both cases the separation by thinning methods is less strict than in Fig 5.

In addition there was a relationship between the ratio of proportions of trees marked by most and by no raters, *P*_m_ / *P*_0_, and the mean conformity number (Fig 6C). Here again we observed inhomogeneity effects caused by the thinning methods (with the aforementioned exception of experiment Loch Ard 2015). There was also a significant relationship between the proportion of trees marked by most raters, *P*_m_, and the mean ratio of the proportion of number and basal area of selected trees, $\bar{B}$ (Fig 6D).

### Summary of tree marking agreement in British forestry

**Fig 7:**
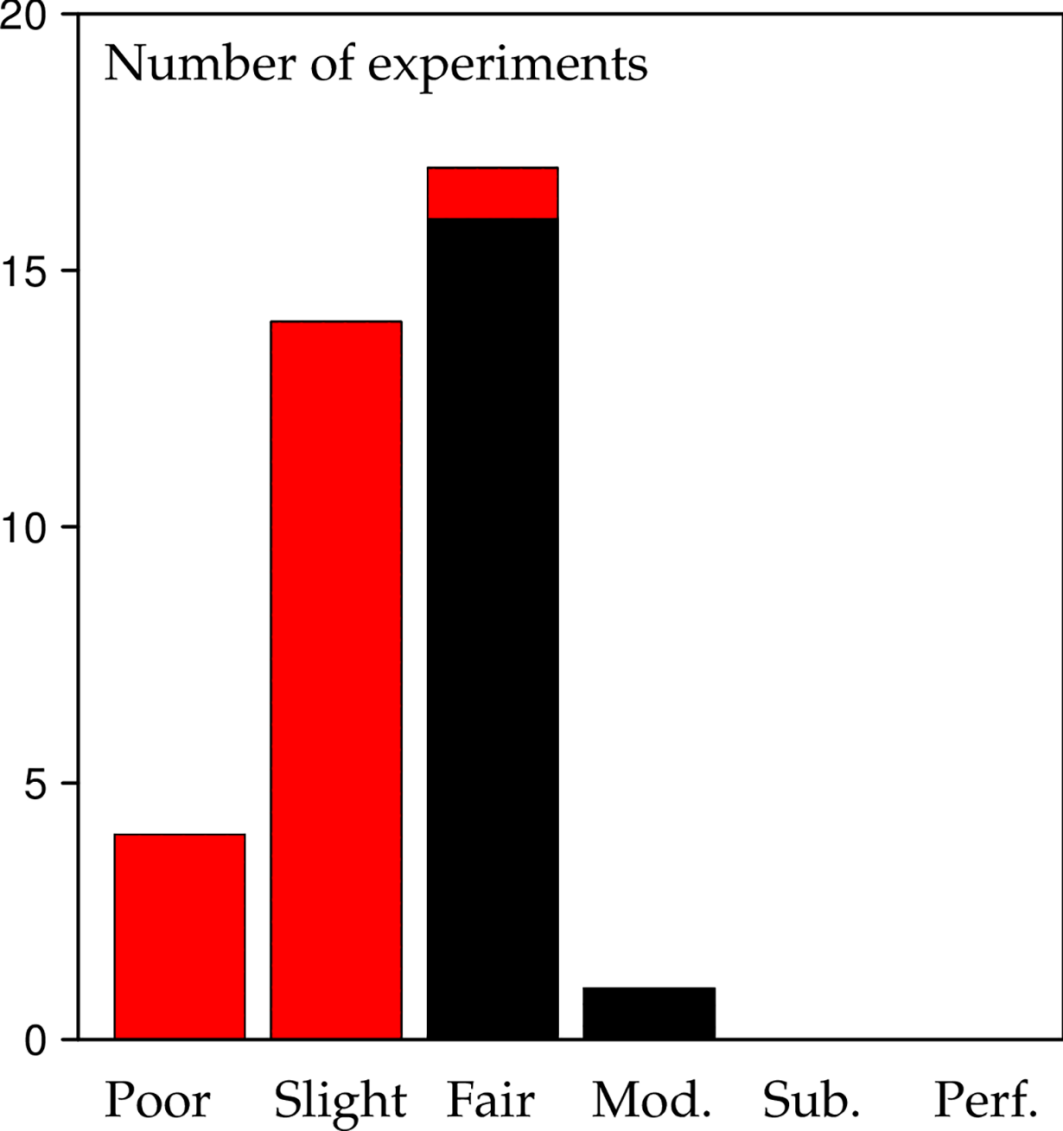
The empirical distribution of *κ*_*F*_ according to the GB data and Table 3. Black–low thinning, red–crown thinning.

We compiled a summary of the 36 experiments by arranging the *κ*_*F*_ values in a bar chart according to Table 3. Fig 7 clearly shows that the crown thinning experiments feature at the lower end of the *κ*_*F*_ distribution and the low thinning experiments at the upper. There was poor to fair agreement in crown thinning experiments and fair to moderate agreement in low thinning experiments. In general, there were no cases of substantial and (almost) perfect agreement, the majority of experiments were between slight and fair agreement.

## Discussion and conclusions

This research involving data from a wide range of sites throughout Britain has highlighted that contrary to textbook and common industry opinion tree marking in forestry generally is not very exact in terms of selecting specific, individual trees. General agreement is only slight to fair (Fig 7) and therefore considerably lower than the kappa values reported in medicine [45]. Despite good education substantial or almost perfect agreement in tree selection is impossible and poor agreement does occur in 4 out of 36 experiments. Independent of management type the variability of selecting individual trees is always considerable, even if good instructions and training are provided. Naturally the question has to be raised in this context whether exact tree marking is even necessary or whether adherence to general trends provided by the instructions suffices. Selecting similar but different trees can also be viewed as a way of reducing the risk of “putting all eggs in one basket”. Future research in this field needs to clarify how much agreement in terms of selecting individual trees is necessary to ensure management objectives.

The study has also clearly shown that forestry staff make different decisions depending on which thinning type they apply. We found consistently larger *κ*_*F*_ values in low-thinning than in crown-thinning experiments. Accordingly agreement is generally considerably higher in low thinnings than in crown thinnings. It is well known that forestry staff in the UK and Ireland are usually more familiar with and better trained in low thinnings [24]. The crown-thinning method is still new and uncommon to many in these countries. This new method, however, is considered as an important part of Continuous Cover Forestry [30]. Internationally this near-natural approach to forest management is on the increase and it is necessary for forestry staff to come to terms with its implications [24]. The crown-thinning method does not inherently lead to less agreement among forestry staff carrying out the same task. Therefore a logical consequence of our research results should be that forestry staff in the UK need more training in crown thinnings. In general terms, a lack of agreement can indicate insufficient information and/or skills.

It may be of interest to return to the curious case of the experiment Loch Ard in 2015, the only case where the raters achieved a high *κ*_*F*_ of 0.44 in a crown-thinning experiment. Investigating the details of this experiment have brought to light that this particular group mainly consisted of forest contractors and that at least 70% of them were machine operators. Their typical professional duties usually do not involve tree selection. As a consequence they probably took the crown-thinning instructions literally for what they were whilst their thinking was not “contaminated” by other practices or past experience. Therefore the raters in this experiment must have entirely focused their attention on selecting larger trees for removal. This explanation is confirmed by the findings in [24].

On a technical side, given our data there is hardly any practical difference between Fleiss’ kappa, *κ*_*F*_, and the Conger-Hubert-Schouten kappa, *κ*_*CHS*_ (Fig 5). This is a very interesting finding: Although the underlying statistical theory suggests a number of advantages [34], the values of the latter are very close to those of the original Fleiss’ kappa. This can be explained by the fact that the rater bar charts all were sufficiently close to a uniform distribution, i.e. all raters rated with quite similar intensities. In that case *κ*_*F*_ is very similar to *κ*_*CHS*_ [34].

A certain lack of agreement as found in this study is usually a good starting point for more detailed analyses: We could demonstrate that the rater and marking bar charts representing the marginal distributions of the rating data matrix offer valuable information about active and passive processes involved. This information was conveyed by the typical shapes of the bar charts and could be synthesised in four parameters, namely *r*_*v*_ and $\bar{c}$ for the rater bar chart and *P*_0_ and *P*_m_ for the marking bar chart. All but *P*_0_ have shown great value in this study, however also the ratio *P*_m_ / *P*_0_ is meaningful.

Interestingly there was no correlation between Fleiss’ kappa, *κ*_*F*_, and measures of forest stand structure. This is probably related to the fact that the sites selected for the marteloscope experiments were quite similar in structure, as they all were intended to capture the situation of somewhat “neglected” plantations at the start of a transformation to continuous cover forestry. However, we were able to identify weak correlations between the mean conformity number $\bar{c}$ and the coefficient of variation of stem diameters and the skewness of the empirical stem diameter distribution, respectively (Fig 6A and 6B). We expected to gain information on rater agreement from this characteristic that kappa does not yield. Indeed, $\bar{c}$ is somehow correlated with *k*_*d*_ and *v*_*d*_ (see Fig 6A and 6B) whilst kappa is not. On the other hand, kappa and $\bar{c}$ are related (see Fig 5C). The mean conformity number can therefore be interpreted as a surrogate measure of Fleiss’ kappa.

Contrary to our initial hypothesis, our results suggest that more complex stem-diameter structures in forests facilitate the decision process, possibly by providing stronger contrasts between possible choices. This is also an interesting finding. For future reference concerning similar meta-studies, it can therefore be recommended to set up marteloscope experiments in more varied stand structures. The discussion of experimental design may also include the question whether subsetting the trees of a certain study area may be a better idea for gathering information on rater behaviour: Such a design could potentially ignore trees contributing to *P*_0_, i.e. obvious cases that are not likely to be selected by any rater and have a stronger focus on trees that are difficult to rate, since it is psychologically easier for humans to agree on negative cases [41]. This could potentially help to elaborate more clearly the difference in the behaviour of different raters, since negative agreements tend to increase the kappa values leading to a pseudo agreement. In practical terms, a group of experimenters could pre-select a subset of trees in a marteloscope that they collectively perceive as difficult to judge on. The subset is not revealed to the raters. In the analysis, the experimenters then analyse the ratings once for all trees and once for the subset alone. This will allow to understand how trees that are difficult to judge on influence the outcome of the experiment.
